# Low-Dose Antenatal Calcium Supplementation: An Intervention Ready for Prime Time

**DOI:** 10.9745/GHSP-D-24-00074

**Published:** 2024-02-28

**Authors:** Stephen Hodgins, Matthews Mathai

**Affiliations:** a Editor-in-Chief, Global Health: Science and Practice Journal; School of Public Health, University of Alberta, Edmonton, Alberta, Canada.; b Associate Editor, Global Health: Science and Practice Journal; Independent consultant, St. John’s, NL, Canada.

## Abstract

New evidence of the effectiveness of low-dose antenatal calcium supplementation for preventing preeclampsia and preterm birth provides additional protection for pregnant women and their newborns in settings where calcium intake is low.

On January 11, 2024, the *New England Journal of Medicine* published an article reporting the results of a non-inferiority study comparing low- vs. high-dose antenatal supplementation in populations with low calcium intake, with the primary endpoints being preeclampsia and preterm birth.^1^ This study has important implications for maternal and newborn health programs in low- and lower-middle-income countries where calcium intake is low.

Preeclampsia/eclampsia (PE/E) is second only to hemorrhage as a direct cause of maternal deaths and is estimated to account for about 14% of maternal deaths.^2^ According to data from a 6-country World Health Organization (WHO) study,^3^ PE/E is the primary obstetrical cause for 24% of perinatal deaths. For decades, there has been evidence of an association between low calcium intake and risk of PE/E.^4^

Trials in the first decade of this century, notably a large 6-country study by WHO,^5^ provided evidence for the efficacy of antenatal calcium supplementation for preventing severe PE/E. The WHO study used a dose of 1.5 g/day of elemental calcium in the form of calcium carbonate. The risk of both severe obstetrical complications and newborn death was reduced by 30%, and risk of early preterm birth (less than 32 weeks’ gestation) was reduced by 18%. A Cochrane review, including the results from the WHO study, found the risk of preeclampsia reduced by 55% and of overall preterm birth by 19%.^6^ Trials of lower-dose calcium (generally 500 mg of elemental calcium), as summarized in the Cochrane review, showed similar results. However, in the aggregate, they were still inadequately powered to constitute robust evidence for efficacy.^6^ Nevertheless, the evidence from the higher-dose trials (generally 1.5 g of elemental calcium) was judged by WHO to be adequate to warrant recommending the use of this intervention in populations with low calcium intake, as reflected in WHO guidance released in 2011.^7^ At that point, we had good evidence of efficacy for an intervention addressing an important public health problem—calcium-deficiency-attributable PE/E—and its associated consequences for mothers and their infants. And we had clear, unequivocal endorsement from WHO. But since then, few countries have added antenatal calcium supplementation to their routine antenatal services.^1^ Why not?

This intervention—at the recommended 1,500–2,000 mg/day dosing—isn’t easy for pregnant women or for health systems. As specified in the 2011 guidance, the supplement is to be taken as a single 500–600 mg tablet at 3 different times a day, spaced out with the usual dose of iron-folate (or multi-micronutrient) supplement to avoid compromising iron absorption. That means taking pills at 4 different times each day.

For the health system, this commodity at this dosing is comparatively expensive and logistically challenging. In the 2016 WHO antenatal care guidelines, it is noted that the cost, based on bulk procurement prices in 2013, would be approximately US$11.50 per pregnancy. By comparison, the cost for iron-folate tablets was less than 1/5 of that amount.^8^ Furthermore, with this dosage regimen, the weight of the tablets required per pregnancy comes to over 1 kg, making it challenging for those charged with managing the government health care commodity supply chain.

Evidence from the newly published article by Dwarkanath and colleagues^1^ provides an opportunity to address these challenges. It reports on trials conducted in India and Tanzania that together enrolled over 20,000 pregnant women, comparing outcomes for 500 mg of elemental calcium per day vs. 1.5 g. This well-conducted, adequately powered study found, in pooled analysis, sound evidence for non-inferiority on its primary outcomes of preeclampsia and preterm birth. Similarly, it did not find a difference between the 2 dosing regimens on any of its secondary outcomes, which included severe preeclampsia, low birth weight, and infant death within 42 days of birth.

There is now adequate evidence that we have an efficacious intervention that can feasibly be made available to pregnant women in settings where calcium intake is low, with fewer adherence challenges, lower costs, and more easily manageable commodity logistics than there has been with high-dose calcium. This simple intervention has the potential to substantially reduce the burden of one of the 2 most important direct causes of maternal death. Furthermore, the original 6-country WHO trial suggests the intervention could reduce the risk of newborn death by up to 30% in populations with low calcium intake.

We are confident that in the coming months, based on this new evidence, WHO will update its current recommendations for antenatal calcium supplementation. It is also time now for governments and their development partners in countries with populations with low calcium intake to begin planning to incorporate this intervention into routine antenatal care.

## Notes

**First Published Online:** February 16, 2024.
